# *IL6 and CRP *haplotypes are associated with COPD risk and systemic inflammation: a case-control study

**DOI:** 10.1186/1471-2350-10-23

**Published:** 2009-03-09

**Authors:** Dilyara G Yanbaeva, Mieke A Dentener, Martijn A Spruit, Jeanine J Houwing-Duistermaat, Daniel Kotz, Valéria Lima Passos, Emiel FM Wouters

**Affiliations:** 1 Department of Respiratory Medicine, Nutrition and Toxicology Research Institute Maastricht (NUTRIM), Maastricht University Medical Centre, Maastricht, The Netherlands; 2 Department of Research, Development & Education, Centre for Integrated Rehabilitation of Organ failure (CIRO), Horn, The Netherlands; 3 Department of Medical Statistics and Bioinformatics, Leiden University Medical Centre, Leiden, The Netherlands; 4 Department of General Practice, School for Public Health and Primary Care (CAPHRI), Maastricht University Medical Centre, Maastricht, The Netherlands; 5 Methodology & Statistics Department, Maastricht University Medical Centre, Maastricht, the Netherlands

## Abstract

**Background:**

Elevated circulating levels of C-reactive protein (CRP), interleukin (IL)-6 and fibrinogen (FG) have been repeatedly associated with many adverse outcomes in patients with chronic obstructive pulmonary disease (COPD). To date, it remains unclear whether and to what extent systemic inflammation is primary or secondary in the pathogenesis of COPD.

The aim of this study was to examine the association between haplotypes of *CRP*, *IL6 *and *FGB *genes, systemic inflammation, COPD risk and COPD-related phenotypes (respiratory impairment, exercise capacity and body composition).

**Methods:**

Eighteen SNPs in three genes, representing optimal haplotype-tagging sets, were genotyped in 355 COPD patients and 195 healthy smokers. Plasma levels of CRP, IL-6 and FG were measured in the total study group. Differences in haplotype distributions were tested using the global and haplotype-specific statistics.

**Results:**

Raised plasma levels of CRP, IL-6 and fibrinogen were demonstrated in COPD patients. However, COPD population was very heterogeneous: about 40% of patients had no evidence of systemic inflammation (CRP < 3 mg/uL or no inflammatory markers in their top quartile). Global test for haplotype effect indicated association of *CRP *gene and CRP plasma levels (P = 0.0004) and *IL6 *gene and COPD (P = 0.003). Subsequent analysis has shown that *IL6 *haplotype H2, associated with an increased COPD risk (p = 0.004, OR = 4.82; 1.64 to 4.18), was also associated with very low CRP levels (p = 0.0005). None of the genes were associated with COPD-related phenotypes.

**Conclusion:**

Our findings suggest that common genetic variation in *CRP *and *IL6 *genes may contribute to heterogeneity of COPD population associated with systemic inflammation.

## Background

Chronic obstructive pulmonary disease (COPD) is a multi-component respiratory disease with recognized systemic impact [1]. Numerous studies performed in recent years provide overwhelming evidence of COPD as a condition characterized by an abnormal inflammatory response beyond the lungs with evidence of low-grade systemic inflammation [2-5]. Raised levels of acute phase proteins like C-reactive protein (CRP), fibrinogen and pro-inflammatory cytokines such as interleukin (IL)-6 were found in circulation of stable COPD patients [3,6] and have been shown to be associated with impaired functional capacity [7], reduced daily physical activity [8] and decreased health status [5,7,9]. However, given the cross-sectional nature of most studies performed so far and possible confounding by a number of lifestyle factors associated with levels of inflammatory biomarkers [10], it is not clear whether these proteins are simply markers of the inflammatory process accompanying chronic diseases such as COPD or key players in the pathogenesis of disease.

Genome-wide scans, twin and family studies have shown that circulating levels of CRP, fibrinogen and IL-6 are heritable (estimated as 25%–40%) [11-15]. Furthermore, recently *CRP *and *FGB *polymorphisms/haplotypes have been described that may partly explain heritability of acute-phase protein and cytokine levels [16-19]. Genetic association testing of genotypes, which influence circulating levels of proteins and directly relate to the outcome of interest, was suggested as more accurate unconfounded estimate of whether systemic inflammation levels causally influence outcome [20].

In the present study we investigate whether common haplotypes in *CRP, IL6 *and *FGB *(encoding fibrinogen β chain) genes influence systemic inflammatory status in COPD, the risk for COPD and, eventually, different disease-related phenotypes. Some of the results of this study have been previously reported in the form of an abstract [21].

## Methods

### Study participants

The investigation was designed as a case-control association study, consisting of unrelated individuals recruited from the same geographical area (Limburg province, the Netherlands). A total of 556 Caucasian subjects were investigated. All subjects were current or former smokers. Three hundred and sixty-one patients with clinically stable moderate-to severe COPD entering pulmonary rehabilitation (Center for Integrated Rehabilitation of Organ failure (CIRO), Horn, The Netherlands) were enrolled for the study. Clinical history of COPD and the degree of the disease severity were assessed according to the published Global Initiative for Chronic Obstructive disease (GOLD) guidelines [22]. One hundred and ninety five healthy (ex-) smokers were recruited as controls. The healthy control subjects were volunteers recruited through advertisement in a local newspaper. Part of the healthy controls were also recruited through the COSMO study [23]. Inclusion criteria for both groups were: Caucasian origin, 40 years of age or older, smoking history of 10 pack-years or more, completed spirometry and blood sample donation. The ethical review board of the University Hospital Maastricht approved the study, and all subjects gave their written informed consent.

### Clinical examination and inflammation measurement

Lung function was determined using spirometry, height and weight were measured in every participant and body mass index was calculated. Several COPD-related clinical characteristics were assessed only in COPD patients based on standard procedures (see Additional file 1). Plasma levels of CRP, IL-6 and fibrinogen were measured by high-sensitivity particle-enhanced immunoassay, ELISA and coagulation reaction respectively. Further details are provided in Additional file 1.

### TagSNP selection and genotype determination

TagSNPs were selected for genotyping from the SeattleSNPs database  using resequencing data from 23 unrelated European Americans. Polymorphisms with a minor allele frequency of less 5% were not included. Six polymorphisms in *CRP*, 8 SNPs in *IL6 *and 6 SNPs in *FGB *were selected. For two non-redundant SNPs of *FGB *the development of the genotyping assay failed (rs2227432, rs2227439), leaving 4 tagSNPs for the analysis. For further details on DNA processing and genotyping see Additional file 1.

### Statistical analysis

#### Descriptive statistics and baseline comparisons

Variables are presented as proportions (percentage), mean ± standard deviation (SD) or median (range) depending on their measurement scale and distribution. Plasma levels of inflammatory mediators were skewed to the right. They were natural logarithmically transformed to achieve symmetric distribution. Baseline differences between groups were analyzed using Student's *t *test and Mann-Whitney test for continuous variables and χ^2 ^square test for categorical variables. Prevalence of systemic inflammation has been accessed by number of inflammatory markers in the top quartile and by CRP levels. The correlations between inflammatory markers and the different clinical phenotype were estimated using Pearson's or Spearman's correlation coefficient. Multiple linear regression models were performed to search for the best predictors of CRP, IL-6 and fibrinogen levels.

#### Genetic association analysis

To determine the linkage disequilibrium (LD) we have calculated the D' statistics between SNPs. Hardy-Weinberg equilibrium for each tagSNP was tested by the exact χ^2 ^statistic. SNP rs2069849 in *IL6 *was excluded from haplotype analysis because of low allele frequency (3%). To reduce the problem of multiple testing we selected haplotypes as major genetic variable. Differences in haplotype distributions were tested using the global statistic of Schaid under additive genetic model [24]. If the global haplotype test achieved a P-value < 0.1, haplotype-specific analysis was performed. Haplotype-specific effects were estimated using linear and logistic regression as implemented in the haplo.stats package in R. Analyses were adjusted for potentially confounding factors (see Additional file 1 for full description). P-values less than 0.05 were considered significant (two-sided).

Eight primary genetic tests were performed. First, each gene was tested for association with two major outcomes: either corresponding protein level or COPD. These comprised 6 tests. Additionally, given that IL-6 is a major regulator of hepatic production of CRP and fibrinogen, we also investigated the association of *IL6 *haplotypes with CRP and fibrinogen plasma levels (2 tests). To reduce the problem of spurious associations due to multiple testing, Bonferroni correction was applied for those tests (*P*_*corrected *_= 0.05/8 tests = 0.006).

Body-Mass Index, Airflow Obstruction, Dyspnea, and Exercise Capacity (BODE) index, 6 minute walking distance (6MWD), maximum workload at the cardiopulmonary test, Medical research council (MRC) score, body mass index (BMI) and forced expiratory volume in 1 sec (FEV1) were tested after initial 8 analyses have been performed and treated as secondary outcomes. Secondary COPD outcomes were not considered for a conservative Bonferonni correction, because of a high degree of correlation with each other.

## Results

### Systemic inflammation

Table 1 summarizes the demographic data and baseline characteristics of study groups. Compared with the healthy smokers, COPD patients were older, more likely to be men and had more pack-years smoking. On average, patients had moderate-to-severe airflow obstruction and clear functional exercise intolerance.

**Table 1:** Baseline characteristics of COPD patients and healthy controls*

*Characteristics*	*Controls* *N = 195*	*COPD* *N = 355*	*P-value*
Years of age	54.3 (7.3)	64.2 (9.4)	<0.001
Male, N (%)	94 (48)	219 (62)	0.002
Current smoker, N (%)	115 (59%)	90 (25%)	<0.001
Tobacco consumption, pack years-smoked	29.6 (14.8)	39.9 (18.5)	<0.001
FEV1, liters	3.2 (0.7)	1.1 (0.5)	<0.001
FEV1,%pred	103.3 (15.1)	41.9 (16.0)	<0.001
FEV1/FVC, %	78.3 (5.3)	42.0 (11.9)	<0.001
FVC, liters	4.1 (0.9)	3.0 (0.9)	<0.001
FVC,%pred	109.3 (16.8)	86.3 (21.1)	<0.001
BMI, kg/m2	26.4 (3.7)	25.0 (5.0)	<0.001
Hypertension^†^, N (%)	33 (17%)	88 (25%)	0.01
PaO2, kPa	-	9.2 (1.37)	
PaCO2, kPa	-	5.5 (0.80)	
Long-term oxygen therapy, N (%)	-	82 (23.43)	
6 minute walking distance, meters	-	420.1 (127.9)	
Maximum workload, watts	-	72.6 (33.9)	
Maximum workload,% pred	-	56.6 (25.2)	
MRC dyspnea score	-	3 (2–4)	
BODE index		4.3 (2.1)	
Systolic blood pressure, mm/Hg	-	135.9 (23.31)	
Diastolic blood pressure, mm/Hg	-	81.1 (13.09)	
Charlson co morbidity index‡	-	1 (1–8)	
CRP, mg/L	1.3 (0.2–3.19)	4.6 (1.3–11.09)	<0.001
IL-6, pg/mL	0.7 (0.45–1.38)	2.1 (1.15–4.24)	<0.001
Fibrinogen, g/L	3.3 (3.00–3.60)	3.6 (3.23–3.82)	<0.001
*Number of plasma markers* *in the top quartile:*			<0.001
0, N (%)	155 (79.4%)	159 (44.5%)	
1, N (%)	29 (14.4%)	88 (24.2%)	
2–3, N (%)	10 (5.2%)	118 (31.3%)	
CRP < 1 mg/L	93 (47.9%)	68 (19.2%)	<0.001
CRP 1–3 mg/L	51 (26.3%)	81 (22.8%)	
CRP 3–10 mg/L	40 (20.6%)	109 (30.7%)	
CRP > 10 mg/L	10 (5.2%)	97 (27.3%)	

*Data presented as mean (standard deviation), median (inter quartile range) or No (%) unless otherwise stated

†Hypertension was defined as blood pressure > 140/90 mmHg or need for antihypertensive treatment.

‡ Median (range)

In general, COPD patients had higher baseline median levels of circulating inflammatory markers (P < 0.001) (Table 1). This difference was still significant after adjustment for age, sex, smoking status, BMI and pack-years smoked. However, the COPD population was very heterogeneous: 27.3% of COPD patients had CRP levels > 10 mg/L, which is a significantly higher proportion than in healthy smokers (5.2%), whereas about 40% of COPD patents had no evidence of systemic inflammation as assessed by CRP levels alone (< 3 mg/L) or by the number of inflammatory markers in their top quartiles (no markers in the top quartile).

Table 2 shows the comparison of clinical characteristics from patients with baseline CRP value < 3 mg/L versus > 3 mg/L. Patients from the "inflammatory" group (CRP > 3 mg/L) were older, more likely to be male, had a higher BMI, more co morbidities and worse results in the 6 minute walking test. Interestingly, besides age and sex, the 6MWD was negatively associated with all 3 inflammatory markers in the backward multiple linear regression analyses (see Additional file 2). Correlations between the inflammatory markers and the functional measures of disease are shown in Additional file 3.

**Table 2:** Comparison of clinical parameters between patients with initial CRP levels ≤3 mg/L versus > 3 mg/L

*Characteristics*	*CRP ≤ 3 mg/L* *N = 146*	*CRP > 3 mg/L* *N = 209*	*P-value*
Years of age	62.1 (8.2)	65.7 (9.8)	< 0.001
Male, N (%)	77 (53%)	142 (68%)	0.004
Current smoker, N (%)	40 (27%)	50 (24%)	0.460
Tobacco consumption, pack years-smoked	40.3 (19.0)	39.6 (18.2)	0.703
FEV1, liters	1.1 (0.5)	1.1 (0.5)	0.922
FEV1,%pred	41.6 (15.9)	41.9 (15.9)	0.846
FEV1/FVC, %	41.0 (11.8)	42.7 (11.8)	0.172
FVC, liters	3.0 (1.0)	3.0 (0.9)	0.884
FVC,%pred	87.5 (22.5)	85.4 (20.3)	0.373
BMI, kg/m2	24.0 (4.4)	25.7 (5.4)	0.001
PaO2, kPa	9.3 (1.5)	9.1 (1.3)	0.259
PaCO2, kPa	5.4 (0.8)	5.5 (0.8)	0.342
Long-term oxygen therapy, N (%)	30 (21%)	52 (25%)	0.205
6 minute walking distance, meters	445.9 (121.2)	401.1 (129.3)	0.001
Maximum workload, watts	77.2 (34.7)	69.2 (33.0)	0.037
Maximum workload,% pred	57.4 (23.6)	56.0 (26.4)	0.636
MRC dyspnea score	2 (2–4)	3 (2–4)	0.108
BODE index	4.1 (2.1)	4.4 (2.1)	0.264
Systolic blood pressure, mm/Hg	134.7 (21.7)	136.8 (24.4)	0.412
Diastolic blood pressure, mm/Hg	80.7 (12.5)	81.4 (13.5)	0.647
Charlson co morbidity index †	1 (1–7)	1 (1–8)	0.007
GOLD stages (%):			
Mild	2.1%	2.4%	0.891
Moderate	27.4%	23.9%	
Severe	35.6%	38.3%	
Very severe	34.9%	35.4%	
CRP, mg/L	1.1 (0.6–1.9)	9.4 (5.8–16.0)	< 0.001
IL-6, pg/mL	1.3 (0.7–2.1)	3.5 (1.6–5.1)	< 0.001
Fibrinogen, g/L	3.3 (3.0–3.6)	3.7 (3.4–4.0)	< 0.001

*Data presented as mean (standard deviation), median (inter quartile range) or No (%) unless stated otherwise

† Median (range)

### Haplotype Tagging SNPs and COPD

From the data of SeattleSNPs, we selected 18 SNPs (Table 3) which together tag the 5 most common (frequency > 2.5%) haplotype groups of *CRP*, the 8 most common haplotype groups of *IL6 *and the 5 most common haplotype groups of *FGB *(Table 4). None of the tagSNPs showed a significant deviation from Hardy-Weinberg in a control group, and the allele frequencies were comparable to those reported in the Seattle SNP panel and other Caucasian populations and individuals of Dutch origin [18,19,25]. All polymorphisms within each gene showed a little evidence of historical recombination as measured by D' (see Additional file 4).

**Table 3:** Tagging SNPs characteristics and single SNP association analysis for COPD

*dbSNP No*.	tagSNP*	*Gene region*	*Minor allele frequency*		*P-value alleles†*	*OR adj‡ genotypes* *(95%CI)*	*P-value genotypes*
			*Controls*	*COPD*			
***IL6 gene *(7p21-p15)**							
rs2069825	205CT/-	5'flanking	0.42	0.37	0.11	0.87 (0.64–1.17)	0.35
rs2069827	321G/T	5'flanking	0.10	0.07	0.10	0.67 (0.39–1.14)	0.14
rs1800797	1086G/A	5'flanking	0.44	0.39	0.12	0.86 (0.64–1.16)	0.32
rs2069840	3437C/G	intron	0.34	0.36	0.52	1.04 (0.76–1.42)	0.81
rs1554606	3572G/T	intron	0.46	0.42	0.19	0.84 (0.62–1.13)	0.25
rs2069849	6021C/T	synonymous	0.03	0.02	0.33	0.51 (0.19–1.36)	0.18
rs2069861	6519C/T	3'flanking	0.11	0.09	0.24	0.93 (0.57–1.52)	0.77
rs1818879	7592G/A	3'flanking	0.32	0.39	0.02	1.49 (1.08–2.04)	0.01
***CRP gene *(1q21-q23)**							
rs3091244	1440C/T	5'flanking	0.30	0.31	0.08	1.01 (0.73–1.40)	0.94
	1440C/A		0.06	0.08		1.57 (0.88–2.79)	0.12
rs1800947	2667G/C	synonymous	0.05	0.08	0.03	1.80 (0.96–3.37)	0.06
rs1130864	3014C/T	3' UTR	0.30	0.31	0.65	1.05 (0.76–1.46)	0.76
rs1205	3872G/A	3'-flanking	0.35	0.33	0.41	0.98 (0.73–1.32)	0.91
rs2808630	5237A/G	3'-flanking	0.29	0.27	0.55	0.83 (0.60–1.15)	0.27
rs3090077	6469A/C	3'-flanking	0.05	0.08	0.15	1.59 (0.86–2.96)	0.14
***FGB gene *(4q28)**							
rs1800791	1038G/A	5' flanking	0.18	0.17	0.63	0.80 (0.55–1.18)	0.26
rs1800788	1643C/T	5' flanking	0.18	0.21	0.31	1.36 (0.94–1.95)	0.10
rs1800787	1744C/T	5' flanking	0.21	0.20	0.59	1.10 (0.75–1.61)	0.62
rs2227421	9952A/C	3' UTR	0.31	0.33	0.59	1.03 (0.74–1.42)	0.87

* TagSNPs are numbered by position in sequences AF449713 for *CRP*, AF372214 for *IL6 *and AF388026 for *FGB*. Base change common allele/rare allele.

† P value is for the test of allele counting in cases versus controls.

‡ Logistic regression analysis was adjusted for age, sex and tobacco consumption (pack-years smoked)

**Table 4:** Frequency distribution in patients and controls and global association tests for haplotypes of *IL6*, *CRP *and *FGB*

*Gene*	Haplotype*	*Controls* *%*	*COPD* *%*	*Global test P-values†*			
				
				COPD	Ln (IL-6)	Ln (CRP)	Ln (fibrinogen)
*IL6*	H1 1111111	17.8	16.1	0.003	0.80	0.08	0.73
	H2 1111112	2.4	6.2				
	H3 1112111	4.8	10.2				
	H4 1112112	27.8	25.0				
	H5 2121211	20.1	16.1				
	H6 2121221	10.4	7.7				
	H7 2221211	10.1	6.3				
*CRP*	H1 111121	28.7	26.6	0.09	Not tested	0.0004	Not tested
	H2 111211	30.3	24.2				
	H3 121211	4.9	8.2				
	H4 212111	29.7	30.4				
	H5 311112	5.4	7.3				
*FGB*	H1 1111	11.9	10.3	0.28	Not tested	Not tested	0.72
	H2 1112	31.2	32.8				
	H3 1121	21.0	19.2				
	H4 1211	18.2	20.9				
	H5 2111	17.4	16.2				

* 1 codes the common allele and 2 codes the minor allele. *IL6 *loci are presented in the order rs2069825, rs2069827, rs1800797, rs2069840, rs1554606, rs2069861 and rs1818879. *CRP *loci are presented in the order rs3091244, rs1800947, rs1130864, rs1205, rs2808630 and rs3090077. *FGB *loci are presented in the order rs1800791, rs1800788, rs1800787 and rs2227421

† Global test p-value from haplo.score for overall association between haplotypes and trait. The COPD analysis was adjusted for age, sex and tobacco consumption (pack-years smoked). Inflammatory markers levels analyses were adjusted for marker-specific clinical covariates: age, sex and 6MWD (all genes) and BMI (only for *CRP*).

Table 3 shows basic characteristics for SNPs and their allele frequency distribution in COPD patients and healthy smokers. Significant difference in allele frequency between patients and controls was found for the *CRP *synonymous SNP 2667G/C (rs1800947) and for the *IL6 *3'UTR SNP 7592G/A (rs1818879). Results of single-SNP analyses performed for all 18 SNPs for each of the major outcome are summarized in Table 3 and Additional file 5. Briefly, an increased risk of COPD was found for carriers of the minor alleles of the rs1800947 and rs1818879. For the tri-allelic *CRP *SNP rs3091244, association with increased CRP levels was observed. We also identified two *CRP *and one *IL6 *SNPs, which were significantly associated with decreased CRP levels (see Additional file 5).

### Haplotype-based analysis, COPD and systemic inflammation phenotypes in COPD patients

Common haplotypes were predicted from genotypic data. First, global test for haplotype association were performed for all 3 genes to provide an overall test with 4 outcomes of interest (Table 4). A significant difference in haplotype distribution between patients and controls was observed for *IL6 *(P = 0.003). There was a trend for association with COPD for *CRP *haplotypes (P = 0.09). At the same time, CRP levels were the most significantly associated with CRP haplotypes (P = 0.0004) and less strongly with *IL6 *haplotypes (P = 0.08) (Table 4).

To investigate the cause of these associations further we calculated haplotype-specific scores and then estimated haplotype effects relative to the most common haplotype when all haplotypes were entered into regression model (Tables 5, 6). In particular, *CRP *haplotype H3 was associated with higher risk of being COPD and low CRP levels when tested against the most common haplotype H4 (Table 5). However, *IL6 *haplotype H2 was associated with even higher decrease in CRP levels (Table 6). Moreover, the *IL6 *haplotype H2 was differently distributed between cases and controls (6.2% versus 2.4%, P = 0.0006) and associated with almost 5 times higher risk of COPD compared to the most common haplotype H4 (OR = 4.82, 95%CI 1.64–4.18, P = 0.004) (Table 6). Thus, the H2 haplotype effect on COPD risk is stronger than the effect of the *IL6 *rs1818879 (OR = 1.49, 95%CI 1.08–2.04) (Table 3).

**Table 5:** Association of *CRP *haplotypes with COPD and ln(CRP)

	COPD*		*Ln(CRP) †*	
Haplotype	OR (95%CI)	P-value	Coefficient ± SE	P-value

H1	0.85 (0.57–1.27)	0.42	-0.33 ± 0.13	0.01
H2	0.86 (0.64–1.16)	0.43	-0.13 ± 0.13	0.34
H3	1.64 (0.83–3.27)	0.16	-0.58 ± 0.20	0.004
H4	REFERENT		REFERENT	
H5	1.35 (0.69–2.65)	0.38	-0.02 ± 0.21	0.92
Others‡	2.91 (0.77–0.98)	0.12	0.66 ± 0.27	0.01

* Logistic regression analyses were adjusted for age, sex and tobacco consumption (pack-years smoked).

† Linear regression analyses were adjusted for age, sex, BMI and 6MWD.

‡ Rare haplotypes (frequency < 2.5%) were coded in aggregate as "others"

**Table 6:** Association of *IL6 *haplotypes with COPD and ln(CRP)

	COPD *		*Ln (CRP) †*	
Haplotype	OR (95%CI)	P-value	Coefficient ± SE	P-value

H1	0.92 (0.56–1.51)	0.75	0.005 ± 0.17	0.98
H2	4.82 (1.64–4.18)	0.004	-0.94 ± 0.27	0.0005
H3	1.71 (0.85–3.44)	0.13	-0.12 ± 0.21	0.54
H4	REFERENT		REFERENT	
H5	1.01 (0.64–1.60)	0.95	0.01 ± 0.17	0.93
H6	0.93 (0.51–1.67)	0.80	-0.36 ± 0.22	0.10
H7	0.71 (0.37–1.36)	0.3	-0.02 ± 0.24	0.94
Others‡	1.48 (0.79–2.78)	0.22	-0.07 ± 0.20	0.73

* Logistic regression analyses were adjusted for age, sex and tobacco consumption (pack-years smoked).

† Linear regression analyses were adjusted for age, sex, BMI and 6MWD.

‡ Rare haplotypes (frequency < 2.5%) were coded in aggregate as "others"

Comparable associations were found when the total group of cases and controls was considered for analysis of systemic inflammation levels (N = 550) (data not shown).

### Genetic variation and COPD-related phenotypes

Given that inflammatory markers were correlated with some disease characteristics (see Additional file 3), we further studied whether corresponding inflammatory genes are associated with important quantitative COPD phenotypes and performed the association analysis for 6 outcomes: BODE index, MRC score, BMI, FEV_1_, maximum workload and 6MWD. For all 3 genes, no significant association was observed when an overall test of association was applied, except for a marginal association of the *CRP *gene and maximum workload (Table 7). Further testing showed borderline association of the haplotype 121211 with maximum workload (P = 0.05).

**Table 7:** COPD-related phenotypes and global association tests (P-values) for haplotypes of *IL6*, *CRP *and *FGB*

	Phenotype*					
	
*Gene*	BODE index	MRC score	6MWD, meters	Maximum workload, Watts	BMI, Kg/m2	FEV1, L
*IL6*	0.38	0.41	0.48	0.81	0.66	0.22
*CRP*	0.33	0.41	0.11	0.06	0.56	0.70
*FGB*	0.74	0.61	0.61	0.64	0.44	0.72

*Global association tests were adjusted for marker-specific clinical covariates selected by backward linear regression. Age and sex were included in all final models.

Other covariates included in regression models per phenotype:

BODE index: smoking status (former/current), P_CO2 _(kPa)

MRC score: FEV_1 _(liters), smoking status (former/current)

6MWD: FEV_1 _(liters), Charlson co morbidity index, diastolic blood pressure, smoking status (former/current).

Maximum workload: FEV_1 _(liters), Charlson co morbidity index, diastolic blood pressure, P_CO2 _(kPa), height.

BMI: FEV_1 _(liters), smoking status (former/current), diastolic blood pressure, PCO2 (kPa)

FEV_1_: Height, systolic and diastolic blood pressure, P_CO2 _(kPa) and P_O2 _(kPa).

## Discussion

Similar to previous studies [4,9], we have shown that circulating levels of CRP, IL-6 and fibrinogen are increased in clinically stable COPD patients comparing to healthy (ex)smokers. Importantly, inflammatory markers distribution has shown a striking heterogeneity of COPD population in relation to the systemic inflammatory response. Furthermore, we observed a significant association between *IL6 *and *CRP *haplotypes and CRP levels in COPD patients as well as in the total study group, similar to the previous population-based and clinical studies [16,26,27]. Surprisingly, in our study those haplotypes were not related to any of COPD functional phenotype including BMI and 6MWD, which were the most strongly correlated with CRP plasma levels similar to the previous reports [4,5,9]. However, when compared to the controls, a new association of *IL6 *gene and COPD was identified. In particular, *IL6 *haplotype H2 (6.2% of cases) was associated with 5 times higher risk of COPD and significant decrease in CRP levels in COPD patients. To our knowledge, the present study is the first haplotype-based association analysis examining common genetic variation in *CRP*, *IL6 *and *FGB *genes on COPD risk and circulating levels of inflammatory markers in COPD patients and healthy smokers.

Although presence of systemic inflammation has been widely accepted in the last years as an essential characteristic of COPD [3,28], our data clearly show that levels of CRP and 2 other widely studied in COPD markers, IL-6 and fibrinogen, vary significantly across the COPD population. This gives us strong evidence that not all COPD patients have a manifested low-grade systemic inflammatory response. In fact, comparable findings was reported in our previous study of CRP in relation to COPD phenotypes [7]. Further evidence of heterogeneity in relation to CRP comes from a recent intervention study [29], which has shown that COPD patients segregate for provastatin-responders (CRP > 1 mg/uL) and non-responders (CRP < 1 mg/L) based on their baseline CRP levels. Those non-responders may represent the distinct COPD subpopulation with low CRP levels which has impaired immune response to bacteria, viruses and other agents. Interestingly, our findings also suggest that low CRP levels may be associated with significantly higher COPD risk in carriers of a specific IL6 haplotype. Given that lung-expressed *CRP *could be up regulated by cytokines and has shown cyto-protective effect in innate immune response against bacteria and particulate matter [30-32], *CRP *and *IL6 *haplotypes should be further studied in relation to the local impairment in COPD.

In previous reports there seems to be inconsistency about the relationship involving biomarkers levels, corresponding genes and COPD phenotypes. On the one hand, significant associations between the plasma levels of CRP, IL-6 and fibrinogen and COPD phenotypes have been observed [2,7,8,33]. Yet, on the other, no relationship between the polymorphisms influencing the protein levels and COPD phenotypes could be demonstrated in the current and some other smaller studies [34,35]. Interestingly, three recent mRNA/protein profiling studies in quadriceps and *vastus lateralis *of COPD patients [36-38] failed to confirm a common hypothesis that increased expression of pro-inflammatory cytokines (IL-6, IL-8, IL-1 and TNF-α) induce muscle atrophy in COPD patients with muscle weakness. Several phenomena might explain differences between studies of inflammatory genes, mRNA and proteins in COPD. It is well-known that results of observational studies of inflammatory markers might be confounded because of residual association with numerous non-diseases related factors (i.e. lifestyle and socioeconomic) [10,39]. Thus, association of *CRP *or *IL6 *variation with disease phenotype may suggest a causal relationship between CRP levels and disease [20]. However, genetic variation in *CRP *actually only explains a relatively small proportion of CRP levels variation (< 3%), compared with other environmental or genetic factors [16,26,40]. Moreover, given the fact that COPD might share systemic inflammatory phenotype with cardiovascular disease, cancer and diabetes [41,42], which are known co morbidities of COPD, conflicting association data may be caused by different composition of the COPD populations used across different studies. Lastly, acute-phase and pro-inflammatory cytokine genes may be only modifiers of other, not widely studied, inflammatory genes, which are critically involved in COPD pathogenesis. Further hypothesis-free genome-wide high-throughput screening and gene expression profiling studies are needed to validate existing and find new candidate markers characterizing distinct clinical profiles of multi-faced COPD population.

Our study has several strengths. It is the first one that has been specifically designed to evaluate relationship between systemic inflammation and COPD on both genetic and protein level. For this purpose, both standard outcomes (COPD diagnosis and lung function) as well as functional disease-related phenotypes (exercise capacity and body composition) were taken into account. We used a tagging SNP based approach capturing all common variability across the genes of interest to investigate whether genetic variation in the genes of interest influences the risk of COPD. Next this study is based on a well-characterized ethnically homogeneous cohort of stable COPD patients and spirometry-proved healthy smokers collected from the same geographic region.

In our analysis of 3 genes in relation to 4 primary outcomes it is possible but unlikely that results were false positives obtained due to a type I error inflation. It is worth mentioning, two associations (*IL6 *haplotypes and COPD, global P = 0.003, *CRP *haplotypes and ln(CRP), global P = 0.0004) were still significant after applying the conservative Bonferroni correction. In addition, these associations remained also significant at P < 0.002 after permutation tests with at least 10000 simulations. Nevertheless, all reported associations between COPD and *CRP *and *IL6 *genes require replication in other independent studies. Similar to our results, three previous studies of the *IL6 *gene could not find any association between COPD and the SNPs rs1800795 and rs1800797 [43-45]. Interestingly, a new association was found with the IL6 SNP rs1800796 in COPD patients from Spain [44]. Because of a low allele frequency in Europeans (4%), this SNP was not selected for current study; however, further studies using large samples size of Dutch COPD patients are required to replicate this new association.

There are also some limitations of the present study. First, the patients with COPD included in the study were assessed to enter pulmonary rehabilitation and had predominantly moderate-to-severe stages of COPD. This could affect generalizability of our results. Next, a relatively small sample size could have underpowered some observed genetic associations. Namely, *IL6 *association with CRP levels and *CRP *association with COPD were marginally significant in the overall testing but have shown strong haplotype-specific effects in further analysis. Furthermore, as a result of the failure of 2 *FGB *tag SNPs for genotyping we were not able to discriminate one common (defined by rs2227439) and one rare (carrying the minor allele of rs2227432) haplotypes of the *FGB *gene [19]. They were non-discriminative from the *FGB *haplotypes 1111 and 1121, respectively.

It is also noticeable, that, although we found an increased risk of COPD for the *IL6 *H2 carriers, the functional SNP causing this risk still has to be identified. *IL6 *haplotype H2 is tagged by the minor allele of rs1818879. However, it is unlikely, that this SNP located in 3'UTR region of *IL6*, is functional because no association between haplotype H4 (tagged by the combination of rs2069840 and rs1818879) and COPD risk was found. Most likely, the functional SNP lies on the *IL6 *H2 haplotype and was not genotyped in our study. Future resequencing of *IL6 *haplotype H2 carriers may help to identify functional SNP(s). Given that a recent genome-wide association study [46] has found a SNP in IL-6 receptor gene as one of the top associated with six lung function phenotypes and the association we found between COPD and *IL6 *gene, it would be worthwhile to study IL-6 pathway further [47].

## Conclusion

In conclusion, *CRP *and *IL6 *haplotypes were shown to be associated with systemic inflammation and COPD but not with exercise capacity, dysnoea, BMI and BODE index. Future studies are needed to replicate the observed associations in large well-defined COPD cohorts.

## Abbreviations

6MWD: Six Minute Walking Distance; BMI: Body Mass Index; BODE: Body-Mass Index, Airflow Obstruction, Dyspnea, and Exercise Capacity Index; CI: Confidence Interval; COPD: Chronic Obstructive Pulmonary Disease; CRP: C-Reactive Protein; dbSNP: NCBI Single Nucleotide Polymorphism Database; FEV_1_: Forced Expiratory Volume in 1 Sec; FGB: Fibrinogen β; FVC: Forced Vital Capacity; IL6: Interleukin 6; LD: Linkage Disequilibrium; MRC: Medical Research Council; N: Number; OR: Odds Ratio; Pa_CO2_: Carbon Dioxide Tension of Arterial Blood; Pa_O2_: Oxygen Tension of Arterial Blood; SNP: Single Nucleotide Polymorphism; UTR: Untranslated Region.

## Competing interests

Dr Yanbaeva has no conflicts of interest to disclose. Dr Dentener has no conflicts of interest to disclose. Dr Spruit has no conflicts of interest to disclose. Dr Houwing-Duistermaat has no conflicts of interest to disclose. Dr. Kotz has no conflicts of interest to disclose. Dr Lima Passos has no conflicts of interest to disclose. Prof Wouters is a member of the scientific advisory boards for GSK, Boehringer Ingelheim, AstraZeneca and Numico and received lecture fees from GSK, AstraZeneca, Boehringer Ingelheim. He received research grants between 2004 and 2007 from GSK, AstraZeneca, Boehringer Ingelheim, Centocor and Numico.

## Authors' contributions

DY participated in the design of the study and control group recruitment, carried out genotyping, performed statistical analysis, was responsible for data integrity and drafted the manuscript. MD participated in the design and coordination of the study, supervised protein measurements, was responsible for the study documentation and helped to draft the manuscript. MS participated in the design of the study, coordinated patient recruitment and database maintenance. JJHD performed and coordinated the genetic statistical analysis. DK coordinated the control group recruitment. VLP performed and coordinated the statistical analysis. EFM conceived the study concept and design and supervised the study. All authors contributed to the manuscript revision, read and approved the final text.

## Pre-publication history

The pre-publication history for this paper can be accessed here:



## Supplementary Material

Additional file 1 **The file has detailed description of the methods used in the study.** Click here for file

Additional file 2 **Clinical predictors of plasma CRP, IL-6 and fibrinogen levels in COPD patients which retained in the best-fit multiple linear regression models.** Click here for file

Additional file 3 **Correlation between inflammatory markers and functional measures of disease** Click here for file

Additional file 4 **Linkage disequilibrium structure at candidate genes.** Click here for file

Additional file 5 **Association of *IL6, CRP *and *FBG *tagSNPs and multivariable adjusted CRP, IL-6 and fibrinogen levels in COPD patients** Click here for file
