# Supplementary material for: IL6 and CRP haplotypes are associated with COPD risk and systemic inflammation: a case-control study
Source: BMC Med Genet. 2009 Mar 9;10:23. doi: 10.1186/1471-2350-10-23 (PMC2660301; doi:10.1186/1471-2350-10-23)
Supplement: Additional file 1 — The file has detailed description of the methods used in the study. [file 1471-2350-10-23-S1.pdf]

DETAILED METHODS

Clinical examination

Forced expiratory volume in 1 sec (FEV_1) and forced vital capacity (FVC) were calculated from the flow-volume curve using standardized spirometry. FEV_1 was also performed after inhalation of a β agonist via a metered dose inhaler. BMI was calculated as weight divided by height2 (kg/m2). Hypertension was defined as blood pressure >140/90 mmHg or need for antihypertensive treatment.

Several clinical characteristics were assessed only in COPD patients. To estimate functional exercise capacity, a six-minute walking distance test was conducted twice: the longest distance was used in further analysis [1]. Exercise performance was measured by incremental bicycle ergometry test which was performed on an electromagnetic braked ergometer (Corival 400, Lode, Groningen, the Netherlands). After a 2 minute resting period and 1 minute unloaded cycling, power was increased every minute by 10 Watts. None of the subjects knew the exercise load and all were encouraged to cycle at 60 revs/min until exhaustion.

To assess self-perceived dyspnea in relation to physical disability, the MRC dyspnea scale has been used [2]. Self-reported co-existing morbidity was assessed using the Charlson comorbidity index. Arterial oxygen and carbon dioxide tensions ($Pa O_2$, $Pa CO_2$) were analyzed with a blood gas analyzer. Additionally, use of long-term oxygen therapy (LTOT) was recorded.

Inflammatory markers measurements

Blood was collected in an evacuated tube containing EDTA (Sherwood Medical, St Louis, Missouri, USA) and immediately placed on melting ice. After centrifugation twice at

3000 rpm for 5-10 min at 4 °C within 2 h of collection, plasma samples and buffy coats were subsequently stored at –70 °C until analysis. In plasma, IL-6 was determined in duplicate by Pelkine high-sensitivity sandwich enzyme-linked immunosorbent assay (ELISA) kits (Sanquin, Amsterdam, the Netherlands). CRP was assessed in duplicate by high-sensitivity particle enhanced immunoassay (COBAS Mira Radiometer, Copenhagen). Fibrinogen was assessed using coagulation analyzer (Sysmex CA-7000; Dade-Behring, Liederbach, Germany) according to the Clauss method and calculated from EDTA to citrate plasma values.

TagSNP selection and genotype determination

TagSNPs were selected for genotyping from the SeattleSNPs database **http://pga.gs.washington.edu/** using resequencing data from 23 unrelated European Americans. The SeattleSNPs uses a clustering approach to bin SNPs with similar r^2 for one threshold (we used a stringent 0.80). Polymorphisms with a minor allele frequency of less 5% were not included. One tag SNP was chosen for each cluster bin giving a priority to those located in the coding region, 5' promoter and 3'-untranslated region. Six polymorphisms in *CRP* (rs3091244, rs1800947, rs1205, rs2808630, rs1130864 and rs3093077), 8 SNPs in *IL6* (rs2069825, rs2069827, rs1800797, rs2069840, rs1554606, rs2069849, rs2069861 and rs1818879), and 6 SNPs in *FGB* (rs1800787, rs2227421, rs1800791, rs1800788, rs2227432, rs2227439) were selected. For two non-redundant SNPs of *FGB* the development of the genotyping assay failed (rs2227432, rs2227439), leaving 4 tagSNPs for the analysis.

DNA was extracted from the buffy coat fraction using the QIAmp Mini Kit (Qiagen, Chatsworth, CA). The tagSNPs were genotyped using Taqman SNP allelic discrimination assays by means of an ABI 7900HT instrument according to the manufacture's specifications (Applied Biosystems, Foster City, CA). DNA samples were processed in 384 well plates. Each plate contained 8 negative controls and 16 genotyping controls with known genotype (4 duplicates of 4 different samples). Average genotyping rate was 99%.

Statistical analysis

Power calculation

The power of study with 355 cases and 195 controls to detect associations was estimated using Quanto[3]. Assuming disease prevalence of 10%, odds ratio of 2.0 and minor allele frequencies 0.05 and 0.48 (the lowest and the highest from the Seattle SNPs reference panel), the case-controls sample afforded an estimated power of 72% and 100%, respectively, at two-sided significance level 0.05.

Excluded and missing data

Cook's distance statistic revealed that 6 COPD patients with extreme levels of inflammatory markers (5 subjects with CRP>70mg/L and 1 subject with IL-6 285 pg/uL) were not influential. However, they were removed from dataset as those individuals were likely to have an unrecognized acute inflammatory process. IL-6 and fibrinogen levels were available in all but one study participants, and CRP levels in 519 subjects (4 patients and 27 controls had very low CRP levels below limit of the CRP assay and were excluded from the association analysis of *IL6* and *CRP* haplotypes with CRP levels.

Prevalence of systemic inflammation

To access the commonness of systemic inflammation by means of 3 plasma markers, for every subject we counted the number of inflammatory biomarkers falling into their top quartile simultaneously. The corresponding 75th percentile values were 7.89 mg/L, 3.46 pg/mL, and 3.77 g/L for CRP, IL-6 and fibrinogen respectively. We also accessed distribution of systemic inflammation according to CRP levels, the most stable and validated systemic biomarker [4]. Cut off points of <1, 1-3, 3-10, >10 mg/L were selected based on results of studies for cardiovascular disease risk [4].

Single SNP analysis

SNP effects have been tested under the additive genetic mode. To test whether SNP was associated with CRP, IL6 and fibrinogen levels or COPD we conducted single-SNP multiple

linear and logistic regression analyses adjusting for specific clinical covariates. Logistic regression analysis was adjusted for several confounding factors such as age, sex and tobacco consumption defined as pack-years based on analysis of our sample. To select clinical predictors of systemic inflammation levels, backward selection was applied to determine statistical significance from the following covariates: tobacco consumption (pack-years smoked), smoking status (former/current), post-bronchodilator FEV1 (%pred), BMI (kg/m^2), hypertension (yes/no). Age and sex were included in all models.

Both logistic and linear regressions have been performed in SPSS 11.0 (SPSS Inc, Chicago, IL, USA). P-values less than 0.05 were considered significant (two-sided).

References

1. **ATS statement: guidelines for the six-minute walk test**. *Am J Respir Crit Care Med* 2002, **166**(1):111-117.
2. Bestall JC, Paul EA, Garrod R, Garnham R, Jones PW, Wedzicha JA: **Usefulness of the Medical Research Council (MRC) dyspnoea scale as a measure of disability in patients with chronic obstructive pulmonary disease**. *Thorax* 1999, **54**(7):581-586.
3. Gauderman WJ: **Sample size requirements for matched case-control studies of gene-environment interaction**. *Stat Med* 2002, **21**(1):35-50.
4. Pearson TA, Mensah GA, Alexander RW, Anderson JL, Cannon RO, 3rd, Criqui M, Fadl YY, Fortmann SP, Hong Y, Myers GL *et al*: **Markers of inflammation and cardiovascular disease: application to clinical and public health practice: A statement for healthcare professionals from the Centers for Disease Control and Prevention and the American Heart Association**. *Circulation* 2003, **107**(3):499-511.
