# Supplementary material for: IL6 and CRP haplotypes are associated with COPD risk and systemic inflammation: a case-control study
Source: BMC Med Genet. 2009 Mar 9;10:23. doi: 10.1186/1471-2350-10-23 (PMC2660301; doi:10.1186/1471-2350-10-23)
Supplement: Additional file 2 — Clinical predictors of plasma CRP, IL-6 and fibrinogen levels in COPD patients which retained in the best-fit multiple linear regression models. [file 1471-2350-10-23-S2.pdf]

Table S1

Clinical predictors of plasma CRP, IL-6 and fibrinogen levels in COPD patients which retained in the best-fit multiple linear regression models*

Predictive parameter	*Coefficient (standard error)*	*95%CI*	*P value*
		**Ln (IL-6)**	
Age	0.029 (0.006)	0.017-0.041	0.001
Sex	0.248 (0.108)	0.036-0.461	0.022
6MWD	-0.002 (0.000)	-0.003 - -0.001	<0.001
		**Ln (CRP)**	
Age	0.011 (0.009)	-0.006-0.029	0.205
Sex	0.382 (0.162)	0.064-0.701	0.019
BMI	0.029 (0.014)	0.001-0.058	0.045
6MWD	-0.002 (0.01)	-0.003-0.000	0.012
		**Ln (fibrinogen)**	
Age	0.004 (0.001)	0.001-0.007	0.005
Sex	0.041 (0.027)	-0.012-0.093	0.126
6MWD	-0.0002 (0.000)	0.000-0.000	0.017

*Following variables were entered into the full models:
age (years), sex (female/male), tobacco consumption (pack-years smoked), smoking status (former/current), post-bronchodilator FEV1 (%pred), BMI (kg/m2), PaO2 (kPa), PaCO2 (kPa), diastolic and systolic blood pressure (mm/Hg), 6MWD (meters), long-term oxygen therapy (yes/no), and Medical research council dyspnea score (points), long-term oxygen therapy (yes/no).

Age and sex were entered in all final models.
