# Supplementary material for: IL6 and CRP haplotypes are associated with COPD risk and systemic inflammation: a case-control study
Source: BMC Med Genet. 2009 Mar 9;10:23. doi: 10.1186/1471-2350-10-23 (PMC2660301; doi:10.1186/1471-2350-10-23)
Supplement: Additional file 3 — Correlation between inflammatory markers and functional measures of disease [file 1471-2350-10-23-S3.pdf]

Table S2

Correlation between inflammatory markers and functional measures of disease

	Ln (IL-6)	*Ln (CRP)*	*Ln (fibrinogen)*
Ln (IL-6)	-	0.49*	-
Ln (fibrinogen)	0.47*	0.53*	-
BODE	0.11†	0.06	0.05
MRC score	0.12†	0.08	0.00
FEV_1 %pred	0.01	0.04	-0.05
6MWD (meters)	-0.30*	-0.17*	-0.19*
BMI (kg/m^2)	0.07	0.13†	0.00
Maximum workload (watts)	-0.14†	-0.10	-0.15*

† P<0.05
* P<0.01
