# Supplementary material for: IL6 and CRP haplotypes are associated with COPD risk and systemic inflammation: a case-control study
Source: BMC Med Genet. 2009 Mar 9;10:23. doi: 10.1186/1471-2350-10-23 (PMC2660301; doi:10.1186/1471-2350-10-23)
Supplement: Additional file 4 — Linkage disequilibrium structure at candidate genes. [file 1471-2350-10-23-S4.pdf]

Table S3

Linkage disequilibrium structure at candidate genes

CRP (pair-wise D' statistic)							
	**Rs3091244 C/T**	**Rs3091244 C/A**	**Rs1800947**	**Rs1130864**	**rs1205**	**Rs2808630**	**Rs3090077**
**Rs3091244 C/T**	.	0.9980	0.8708	0.9743	0.9863	0.9416	0.9961
**Rs3091244 C/A**	.	.	0.9916	0.6325	0.9182	0.8865	0.9588
**Rs1800947**	.	.	.	0.9980	0.9991	0.9978	0.9833
**Rs1130864**	.	.	.	.	0.9996	0.9991	0.9962
**Rs2808630**	.	.	.	.	.	0.9992	0.9965
**Rs2808630**	.	.	.	.	.	.	0.9957
**Rs3090077**	.	.	.	.	.	.	.

IL6 (pair-wise D' statistic)							
	**Rs2069825**	**Rs2069827**	**Rs1800797**	**Rs2069840**	**Rs1554606**	**Rs2069861**	**Rs1818879**
**Rs2069825**	.	0.9989	0.9666	0.9633	0.9655	0.9988	0.6508
**Rs2069827**	.	.	0.9988	0.998	0.9988	0.9927	0.6277
**Rs1800797**	.	.	.	0.9569	0.9568	0.9988	0.6819
**Rs2069840**	.	.	.	.	0.9996	0.9979	0.6401
**Rs1554606**	.	.	.	.	.	0.9987	0.7124
**Rs2069861**	.	.	.	.	.	.	0.6002
**Rs1818879**	.	.	.	.	.	.	

FGB (pair-wise D' statistic)				
	**Rs1800791**	**Rs1800788**	**Rs1800787**	**Rs2217421**
**Rs1800791**	.	0.9980	0.9979	0.9986
**Rs1800788**	.	.	0.9336	0.9740
**Rs1800787**	.	.	.	0.9992
**Rs2217421**	.	.	.	.
