# Supplementary material for: IL6 and CRP haplotypes are associated with COPD risk and systemic inflammation: a case-control study
Source: BMC Med Genet. 2009 Mar 9;10:23. doi: 10.1186/1471-2350-10-23 (PMC2660301; doi:10.1186/1471-2350-10-23)
Supplement: Additional file 5 — Association of IL6, CRP and FBG tagSNPs and multivariable adjusted CRP, IL-6 and fibrinogen levels in COPD patients [file 1471-2350-10-23-S5.pdf]

Table S4

Association of *IL6*, *CRP* and *FBG* tagSNPs and multivariable adjusted CRP, IL-6 and fibrinogen levels in COPD patients

SNP	Ln(IL6)*		Ln (CRP) †		Ln (Fibrinogen) ‡	
	Difference (95%CI)	P- value	Difference (95%CI)	P- value	Difference (95%CI)	P- value
IL6						
rs2069825	0.03 (-0.08 - 0.14)	0.56	0.01 (-0.16 - 0.18)	0.9	0.02 (0.00 - 0.05)	0.08
rs2069827	-0.01 (-0.21 - 0.19)	0.93	0.11 (-0.20 - 0.43)	0.49	0.03 (-0.02 - 0.07)	0.2
rs1800797	0.04 (-0.06 - 0.15)	0.43	0.01 (-0.16 - 0.18)	0.9	0.03 (0.00 - 0.05)	0.03
rs2069840	-0.04 (-0.16 - 0.07)	0.49	0.04 (-0.14 - 0.23)	0.64	-0.01 (-0.04 - 0.01)	0.38
rs1554606	0.02 (-0.09 - 0.13)	0.70	-0.01 (-0.19 - 0.16)	0.87	0.02 (-0.01 - 0.04)	0.13
rs2069861	-0.04 (-0.22 - 0.15)	0.70	-0.32 (-0.62 - -0.03)	0.03	0.01 (-0.04 - 0.05)	0.79
rs1818879	-0.01 (-0.13 - 0.10)	0.81	-0.08 (-0.25 - 0.10)	0.38	-0.00 (-0.03 - 0.02)	0.81
CRP						
rs3091244 C/T	Not tested		0.18 (-0.00 - 0.36)	0.056	Not tested	
rs3091244 C/A	Not tested		0.37 (0.06 - 0.69)	0.021	Not tested	
rs1800947	Not tested		-0.35 (-0.67 - -0.02)	0.037	Not tested	
rs1130864	Not tested		0.18 (-0.01 - 0.36)	0.062	Not tested	
rs1205	Not tested		-0.08 (-0.25 - 0.10)	0.39	Not tested	
rs2808630	Not tested		-0.20 (-0.38 - -0.01)	0.038	Not tested	
rs3090077	Not tested		0.33 (-0.02 - 0.67)	0.063	Not tested	
FGB						
rs1800791	Not tested		Not tested		-0.01 (-0.04 - 0.03)	0.74
rs1800788	Not tested		Not tested		-0.03 (-0.06 - -0.00)	0.03
rs1800787	Not tested		Not tested		0.01 (-0.02 - 0.05)	0.36
rs2227421	Not tested		Not tested		0.02 (-0.00 - 0.05)	0.11

*Linear regression analysis for outcome ln(IL-6) were adjusted for age, sex and 6MWD

†Linear regression analysis for outcome ln(CRP) were adjusted for age, sex, BMI and 6MWD

‡Linear regression analysis for outcome ln(fibrinogen) were adjusted for age, sex and 6MWD
